# Reaching priority populations with different HIV self-testing distribution models in South Africa: an analysis of programme data

**DOI:** 10.1186/s12879-025-10662-7

**Published:** 2025-02-25

**Authors:** Mohammed Majam, Karin Hatzold, Webster Mavhu, Angela Tembo, Vincent Zishiri, Jane Phiri, Donaldson Conserve, Zelalem Haile, Thato Chidarikire, Cheryl C. Johnson, Sangiwe Moyo, Gesine Meyer-Rath, Francois Venter

**Affiliations:** 1 Ezintsha, a division of the Wits Health Consortium, University of the Witwatersrand, Johannesburg, South Africa; 2 Population Services International, Cape Town, South Africa; 3 Centre for Sexual Health and HIV/AIDS Research (CeSHHAR) Zimbabwe, Harare, Zimbabwe; 4 Department of International Public Health, Liverpool School of Tropical Medicine, Liverpool, UK; 5 Department of Prevention and Community Health, Milken Institute School of Public Health, George Washington University, Washington, DC USA; 6 Department of Social Medicine, Ohio University Heritage College of Osteopathic Medicine, Dublin, OH USA; 7 HIV Prevention Department, National Department of Health, Pretoria, South Africa; 8 Global HIV, Hepatitis and STI Programmes, World Health Organisation, Geneva, Switzerland; 9 Department of Internal Medicine, Faculty of Health Sciences, University of the Witwatersrand, Johannesburg, South Africa; 10 School of Public Health, Boston University, Boston, MA USA

**Keywords:** HIV self-testing, Community-based, Facility-based, Workplace, Private sector, Key populations, South Africa

## Abstract

**Background:**

As in much of sub-Saharan Africa, substantial HIV testing gaps remain in South Africa, particularly among adult men ages 20–35, young people ages 15–24 and key populations. Innovative strategies, such as HIV self-testing (HIVST), are needed to reach such under-served populations. We evaluated a range of HIV self-test kit distribution models’ potential to reach adult men, young people and key populations in South Africa, to inform targeted approaches.

**Methods:**

This cross-sectional study used data from community and facility-based HIV self-test kit distribution models implemented from October 2017 to April 2020. Self-test kits were distributed as part of the Unitaid-funded Self-Testing AfRica (STAR) programme. Data were collected from individuals who obtained self-test kits through five distribution models. Frequencies and proportions were used to describe the characteristics of the study populations and self-test kit distribution approaches.

**Results:**

Over 2.5 years, 1 071 065 self-test kits were distributed across the five models. Community-based distribution accounted for 63% of total kits distributed, while the private sector (primarily workplace) accounted for 26%. Distribution at public sector health facilities accounted for 7% and distribution through the key population and secondary distribution models accounted for 2% each. Of those obtaining kits, and for whom we collected previous testing data (*n* = 771 612, 72%), 11% had never tested for HIV, 29% had not tested for at least a year, 41% had tested within the last 4–12 months and 19% had tested within the preceding three months. More men (64%) than women obtained self-test kits across all distribution models. The majority (80%) of men obtaining self-test kits were aged 20–40 years, and primarily received these at public transport terminals (36%), workplaces (18%) and hotspots (14%). A small proportion of men was reached through female sex workers.

**Conclusions:**

This analysis of programme data enabled us to identify HIV self-test kit distribution models that are best suited to reach specific priority and under-tested populations, particularly adult men and young people. Models/sub-models that reach self-test users where they live, work and spend time, are likely to result in higher HIVST uptake. Study findings can inform future HIVST scale-up in South Africa.

## Background

Global (95–95-95) targets stipulate that 95% of all people living with the Human Immunodeficiency Virus (HIV) know their HIV status, 95% of all people with diagnosed HIV infection are on sustained treatment and, 95% of all people on HIV treatment are virally suppressed [1]. Several countries in sub-Saharan Africa (SSA), the epicentre of the HIV epidemic [2], are on track to achieve these targets, demonstrating that they are realistic. Aggregated data however mask substantial variations between both geographic areas and population groups [3, 4]. For example, key populations, including female sex workers (FSWs), men who have sex with men (MSM) and people who inject drugs; young people (15–24 years-old) and; adult general population men ages 20–35 – all groups at greatest risk of HIV acquisition – continue to lag behind at each stage of the HIV treatment (95–95-95) cascade [4–7].

Recent global estimates show that compared to women, 1 million, 1.8 million and 1.6 million more men living with HIV do not know their status, know their status but are not on treatment and are not virally suppressed, respectively [8]. Lower uptake of HIV testing and treatment among men in SSA is often due to poor utilisation of public sector health facilities, reflecting both structural and social health systems barriers [9–11]. Social norms around masculinity that emphasise toughness also contribute to an avoidance of health services [9, 11, 12]. Further, greater formal and informal employment among men compared to women hinder access due to job insecurity and high indirect and opportunity costs [6, 11, 13].

Young people experience unique barriers to standard HIV testing services (HTS) that contribute to low testing uptake. These include stigma around HIV testing, fear of disrespect by healthcare providers, concerns over confidentiality and issues of parental/guardian consent [9]. Likewise, key population groups experience pervasive and multi-factorial structural barriers to conventional HIV testing services (HTS), including community- and institutional-level stigma and discrimination [8, 14–16].

Similar to other SSA settings, variations in HIV testing and treatment access exist in South Africa, home to the highest number of people living with HIV globally [2, 17]. As elsewhere, a substantial HIV testing gap remains, particularly among men in general [9–11], young people and key populations including FSWs and MSM [8]. For example, evidence from district-level data in South Africa showed that MSM and FSWs living with HIV were consistently less likely to know their HIV-positive status than the general population. Innovative solutions and decentralised HTS are therefore required to get to these hard-to-reach and chronically under-tested populations. This is particularly so given a new set of ambitious but achievable targets for 2025 [18], and renewed commitment to target specific age and gender groups, key populations and geographic areas to ensure all population groups are equitably served by HIV prevention and treatment services to ultimately end AIDS by 2030 [18, 19].

HIV self-testing (HIVST), whereby someone performs the test and interprets the result themselves, was until 2020 not widely available in SSA due to concerns about accuracy, acceptability, feasibility, safety and costs [20]. It has now been recommended by WHO as an additional approach to increase HIV testing uptake following substantial evidence that it is a safe, acceptable and effective approach to increase access to, and uptake of, HIV testing and subsequently, treatment, care and prevention services [21–30]. HIVST is showing potential to overcome some of the barriers to conventional HIV testing services, including among under-tested populations such as young people, men and key populations in SSA [9, 31–34].

Here, we present data from the Population Services International and Ezintsha (Wits Reproductive Health and HIV Institute, South Africa) Self-Testing AfRica (STAR) initiative (October 2017 to April 2020). The initiative assessed the potential of varying HIV self-test kit distribution models and sub-models to reach adult men ages 20–35, young people (15–24 years-old) and key populations in South Africa. These data would inform targeted approaches needed to reach the remaining 12% of people with HIV in South Africa who are still unaware of their status [5].

## Methods

In 2017, consortium partners in the second phase of the STAR initiative [35] conducted HIVST implementation research in Eswatini, Lesotho and South Africa through different distribution models, including workplace-, facility- and community-based approaches [36]. Distribution models were identified through various STAR consultative processes (e.g., feasibility studies, discreet choice experiments) [16, 37, 38]. Distribution approaches were adapted to the specific target populations (e.g., adult men, young people) to be reached. In South Africa, HIVST was also introduced into the private sector and into existing key populations programmes.

### Description of distribution models and sub-models

HIVST implementation was through five self-test kit distribution models: community-based, private sector, public sector facility, secondary-based and distribution specifically targeting key populations. Three models comprised discrete distribution sub-models (community based, *n* = 4; private sector, *n* = 5 and secondary-based, *n* = 2). Table 1 contains a detailed description of the distribution models/sub-models and their implementation. Of note, some sub-models (e.g., ANC, transport hub (public transport terminal), workplace) were intentionally designed to target adult men 20–35 years-old, the age group at greatest risk of HIV acquisition in South Africa and other SSA settings [6, 9, 11]. All implementation as of December 2020 was subject to COVID-19 adaptations that occurred in the local context.

**Table 1:** Description of models and sub-models

Model	Sub-model	Description
**Community-based distribution**	**Hotspots**	HIV self-test kits were offered to high-risk populations at hotspots through fixed pop-up sites. Hotspots include high foot traffic areas (e.g., busy walkways, shopping centres). After a trained distributor demonstrated self-test kit use, clients either took the kit home or used it onsite, on their own or with the distributor’s assistance. Clients opting to test onsite were given the opportunity to confirm any reactive results
	**Transport hub**	This sub-model’s description is the same as that for the hotspot one, with a specific focus on distribution in public transport terminals, including taxi ranks and train stations
	**Door-to-door**	Within a specific mapped community or geographical location, trained distributors and/or counsellors moved from one household to another, offering HIV self-test kits to eligible clients. Clients either used the kit on their own or with the distributor’s assistance. Clients who self-reported a reactive result were offered the option to confirm their result onsite or at a nearby facility
	**Integrated HTS (mobile)**	This sub-model involved integrating HIVST into existing mobile HIV testing activities. Clients were offered a choice between HIVST and a blood-based rapid diagnostic test (RDT). Clients were given the opportunity to confirm any reactive results
**Private sector distribution**	**General practice (GP) and nurse-led clinics**	Clients attending health services at GP practice or nurse-led clinics were offered HIV testing using HIVST during their consultation. After a demonstration, clients were offered private testing space within the facility to self-test and were asked to self-report their result. Confirmatory testing and ART initiation were available onsite
	**Pharmacy**	Pharmacists and pharmacist assistants at private pharmacies offered self-test kits to clients ≥ 17 years old seeking services that might suggest HIV risk, including emergency contraception, STI treatment, condoms, lubricants and sexual performance enhancers. After a demonstration, clients could choose to either test onsite or take the kit home. Clients who chose to test onsite were given an opportunity to self-report their result to the pharmacist/pharmacist assistant. All who self-reported a reactive result were offered onsite confirmatory testing
	**Workplace**	This sub-model involved distributing self-test kits in formal workplaces (mining, manufacturing, construction, petroleum, agriculture, security sectors). After a demonstration, clients could choose to either test onsite or take the kit home
	**Workplace Associated Communities**	This sub-model involved kits distribution within communities associated with, or directly surrounding, key workplace sectors such as mining and agriculture. For this sub-model, partners and families of employees were offered tests through outreach programmes. Access to these communities was granted in partnership with the companies and community leaders
**Public sector facility distribution**	**Outpatient Department (OPD)**	Within the hospital outpatient waiting areas, distributors created awareness and provided information on both traditional RDT and HIVST. Clients were then offered the choice to self-test onsite using either a blood or oral-based HIVST kit or having blood-based RDT with a counsellor. Confirmatory testing and ART initiation were available onsite
**Secondary-based distribution**	**Antenatal care (ANC)**	After a demonstration and social harm assessment, all first visit ANC attendees were offered self-test kits to take home to support testing of their male sexual partners. A woman could take a HIVST kit for herself to support home disclosure. Consenting women were followed up by telephone or at next visit to acquire information on whether HIVST kit had been used by partner(s)
	**Index testing**	HIVST was offered to newly diagnosed HIV-positive clients (ART clinic) to facilitate partner notification. Telephonic follow-up of HIV-positive index was done to ascertain index partner uptake of HIVST
**Key population distribution**	**Sex worker**	This sub-model was implemented alongside the Wits Reproductive Health and HIV Institute sex worker programme, which provides various health services to sex workers (SW) at static clinics and at outreach sites. SW were offered up to five self-test kits to take for their network, which would either be their non-client sexual partners, their clients, other SW not accessing HTS, a family member or friend considered by the sex worker to be at elevated risk of HIV. All consenting SW were telephonically followed up to determine usage by their networks. Secondary distribution to male clients and partners was also explored in this sub-model

### Data collection and management

Community health workers and distributors collected data using paper-based forms which were specific to each distribution model/sub-model. Each form included a minimum set of parameters including: age, gender, time since last HIV test, whether the test was assisted or unassisted, and whether the test kit was for a primary or secondary recipient. All data were transcribed onto an electronic case report form by a team of data entry clerks using REDCap, and stored on a secure password-protected server.

### Data analysis

We used frequencies and percentages to describe the characteristics of HIV distribution and the demographic characteristics of self-test kit recipients by distribution model and sub-model. All analyses were performed using SAS version 9.4 (SAS Institute, Cary, NC).

## Results

### Characteristics of self-test kit distribution and recipients

A total of 1 071 065 self-test kits were distributed across the models/sub-models (Table 2). Nearly all distributed kits (99%) were oral-fluid-based, with the remainder (1%) being blood-based/finger prick. Self-test kit distribution peaked in 2018 and 2019 but dropped in 2020 due to lower targets during programme wind-down. The community-based distribution model accounted for nearly two-thirds (63%) of total kits distributed, with the highest number of self-test kit recipients reached through transport hubs (42%) and hotspots (18%) sub-models, while the private sector accounted for just over a quarter (26%) of distributed kits, largely through the workplace (23%) sub-model. Distribution through the public sector facility, secondary (ANC & index testing) and key population models accounted for 7%, 2% and 2%, respectively (Table 2). The least number of self-test kits, less than 1% each, were distributed through: Community-based: integrated HTS (mobile) (0.3%) as well as three Private sector sub-models: nurse-led (0.8%), pharmacy (0.6%) and general practitioner (GP) practice (0.04%) (Table 2).

**Table 2:** Characteristics of self-test kit distribution (*N* = 1 071 065)

Model	n (%)
Community-based	675791 (63.1)
Private sector	280505 (26.2)
Public sector facility	70785 (6.6)
Secondary (Antenatal care and Index testing)	21154 (2.0)
Key Population	22830 (2.1)
**Sub-model**	
Community-based: Integrated HTS (mobile)	3219 (0.3)
Community-based: Transport Hub	452316 (42.2)
Community-based: Door-to-door	22888 (2.1)
Community-based: Hotspots	197368 (18.4)
Private Sector: Workplace	243566 (22.7)
Private Sector: Workplace Associated Communities	21881 (2.0)
Private Sector: Pharmacy	6430 (0.6)
Private Sector: Nurse-led	8172 (0.8)
Private Sector: GP Practice	456 (0.04)
Public Sector Facility: Direct distribution (OPD)	70785 (6.6)
Secondary: Antenatal care	14482 (1.4)
Secondary: Index testing	6672 (0.6)
Key Population: Sex Worker	22830 (2.1)
**Test Type**	
Blood-based	11825 (1.1)
Oral-fluid-based	1059240 (98.9)
**Year**	
2017	8098 (0.8)
2018	567573 (53.0)
2019	395268 (36.9)
2020	100126 (9.3)

### Self-test kit recipients’ gender, age by model/sub-model

We analysed characteristics of self-test kit recipients across and within models and sub-models. More males (64%) than females obtained self-test kits across nearly all the five distribution models (Table 3). The exception was public sector facility outpatient department (OPD) where more females obtained self-test kits. Across all five distribution models, the majority (80%) of males obtaining self-test kits were aged 20–40 years (Table 3). The sub-models with the highest proportions of males ages 20–40 were transport hub (36%), workplace (18%) and hotspots (14%) (Table 4). Of note, a small proportion of men (1%) was reached through female sex workers (Table 3).

**Table 3:** Demographic characteristics of self-test kit recipients by distribution model (*N* = 1 071 065)

	**Community-based**	**Key Population**	**Private Sector/workplace**	**Public Sector Facility (OPD)**	**Secondary: ANC & Index Testing**
**Total distributed**	675791 (63.1%)	22830 (2.1%)	280505 (26.2%)	70785 (6.6%)	21154 (2.0%)
**Age**					
12–14	294 (0.0)	35 (0.2)	65 (0.0)	608 (0.9)	80 (0.4)
15–19	21872 (3.2)	744 (3.3)	45461 (1.6)	6393 (9.0)	615 (2.9)
20–24	149119 (22.1)	3125 (13.7)	59126 (21.1)	12826 (18.1)	3480 (16.5)
25–29	183526 (27.2)	5741 (25.1)	62157 22.2)	13415 (19.0)	3216 (15.2)
30–34	141408 (21.0)	5644 (24.7)	58477 (20.8)	10921 (15.4)	3946 (18.7)
35–39	89669 (13.3)	4403 (19.3)	41058 (14.6)	7980 (11.3)	4013 (19.0)
40–44	43215 (6.4)	2102 (9.2)	25887 (9.2)	5103 (7.2)	3007 (14.2)
45–49	24997 (3.7)	572 (2.5)	14279 (5.1)	3645 (5.1)	1347 (6.4)
50 +	19565 (2.9)	362 (1.6)	13690 (4.9)	8824 (12.5)	893 (4.2)
^a^Missing	2126 (0.3)	102 (0.4)	1270 (0.5)	1070 (1.5)	557 (2.6)
**Sex**					
Female	218281 (32.3)	11047 (48.4)	102595 (36.6)	45018 (63.6)	2730 (12.9)
Male	450851 (66.7)	11279 (49.4)	177433 (63.3)	25308 (35.8)	17996 (85.1)
Transgender/other	1380 (0.2)	151 (0.7)	98 (0.0)	24 (0.0)	20 (0.1)
^a^Missing	5279 (0.8)	353 (1.6)	379 (0.1)	435 (0.6)	408 (1.9)
**Previous Testing**					
0–3 months	97314 (14.4)	684 (3.0)	27209 (9.7)	19350 (27.3)	1608 (7.6)
4–12 months	208144 (30.8)	822 (3.6)	79944 (28.5)	28077 (39.7)	2750 (13)
> 12 months	123670 (18.3)	548 (2.4)	82468 (29.4)	14846 (21)	994 (4.7)
Never tested	54063 (8.0)	137 (0.6)	22721 (8.1)	5480 (7.7)	783 (3.7)
^a^Missing	192600 (28.5)	20639 (90.4)	68163 (24.3)	3032 (4.3)	15019 (71.0)

^a^Missing data age (*n* = 5125); sex (*n* = 6854); previous testing (*n* = 299453)

**Table 4:** Demographic characteristics of self-test kit recipients by distribution sub-model (*N* = 1 071 065)

	**ANC**	**OPD**	**Door-to-door**	**Hotspots**	**GP Practice**	**Index testing**	**Integrated HTS (mobile)**
**Total distributed**	14482 (1.4%)	70785 (6.6%)	22888 (2.1%)	197368 (18.4%)	456 (0.0%)	6672 (0.6%)	3219 (0.3%)
**Age**							
12–14	0 (0.0)	608 (0.9)	6 (0.0)	85 (0.0)	0 (0.0)	78 (1.2)	13 (0.4)
15–19	199 (1.4)	6393 (9.0)	985 (4.3)	10791 (5.5)	18 (4.0)	138 (2.1)	259 (8.0)
20–24	1640 (11.3)	12826 (18.1)	4338 (19.0)	45492 (23.0)	57 (12.5)	361 (5.4)	663 (20.6)
25–29	4027 (27.8)	13415 (19.0)	5155 (22.5)	47691 (24.2)	100 (21.9)	1038 (15.6)	734 (22.8)
30–34	4042 (27.9)	10921 (15.4)	4458 (19.5)	36658 (18.6)	96 (21.1)	1502 (22.5)	579 (18.0)
35–39	2661 (18.4)	7980 (11.3)	3102 (13.6)	24409 (12.4)	62 (13.6)	1473 (22.1)	381 (11.8)
40–44	1048 (7.2)	5103 (7.2)	1935 (8.5)	14778 (7.5)	56 (12.3)	983 (14.7)	240 (7.5)
45–49	310 (2.1)	3645 (5.1)	1044 (4.6)	8153 (4.1)	32 (7.0)	553 (8.3)	151 (4.7)
50 +	123 (0.8)	8824 (12.5)	1162 (5.1)	8266 (4.2)	28 (6.1)	440 (6.6)	197 (6.1)
Missing	432 (3.0)	1070 (1.5)	703 (3.1)	1045 (0.5)	7 (1.5)	106 (1.6)	2 (0.1)
**Gender**							
Female	0 (0.0)	45018 (63.6)	8052 (35.2)	88067 (44.6)	254 (55.7)	1578 (23.7)	1306 (40.6)
Male	14248 (98.4)	25308 (35.8)	14545 (63.6)	107941 (54.7)	200 (43.9)	4947 (74.2)	1906 (59.2)
Transgender/other	0 (0.0)	24 (0.0)	20 (0.1)	129 (0.1)	0 (0.0)	2 (0.0)	1 (0.0)
Missing	234 (1.6)	435 (0.6)	271 (1.2)	1231 (0.6)	2 (0.4)	145 (2.2)	6 (0.2)
**Previous Testing**							
0–3 months	0 (0.0)	19350 (27.3)	1706 (7.5)	15775 (8.0)	55 (12.1)	3 (0.0)	283 (8.8)
4–12 months	0 (0.0)	28077 (39.7)	5301 (23.2)	63841 (32.3)	161 (35.3)	0 (0.0)	1520 (47.2)
> 12 months	0 (0.0)	14846 (21.0)	5990 (26.2)	44372 (22.5)	139 (30.5)	0 (0.0)	783 (24.3)
Never tested	0 (0.0)	5480 (7.7)	4071 (17.8)	8791 (4.5)	35 (7.7)	0 (0.0)	211 (6.6)
Missing	14482 (100)	3032 (4.3)	5820 (25.4)	64589 (32.7)	66 (14.5)	6669 (100)	422 (13.1)
	**Nurse-led**	**Pharmacy**	**Sex Worker**	**Transport Hub**	**Workplace**		**Workplace Associated Communities**
**Total distributed**	8172 (0.8%)	6430 (0.6%)	22830 (2.1%)	452316 (42.2%)	243566 (22.7%)		21881 (2.0%)
**Age**							
12–14	59 (0.7)	3 (0.0)	37 (0.2)	189 (0.0)	3 (0.0)		1 (0.0)
15–19	512 (6.3)	173 (2.7)	777 (3.4)	11019 (2.4)	3844 (1.6)		1097 (5.0)
20–24	1138 (13.9)	968 (15.1)	3285 (14.4)	96699 (21.4)	34726 (14.3)		4164 (19.0)
25–29	1619 (19.8)	1470 (22.9)	6105 (26.7)	127542 (28.2)	59051 (24.2)		5108 (23.3)
30–34	1237 (15.1)	1336 (20.8)	5993 (26.3)	98363 (21.7)	55907 (23.0)		4304 (19.7)
35–39	1050 (12.8)	976 (15.2)	3322 (14.6)	60575 (13.4)	39023 (16.0)		2827 (12.9)
40–44	692 (8.5)	535 (8.3)	2216 (9.7)	25304 (5.6)	24662 (10.1)		1762 (8.1)
45–49	524 (6.4)	290 (4.5)	607 (2.7)	15006 (3.3)	13466 (5.5)		1059 (4.8)
50 +	1072 (13.1)	313 (4.9)	379 (1.7)	17246 (3.8)	12303 (5.1)		1547 (7.1)
Missing	269 (3.3)	366 (5.7)	109 (0.5)	373 (0.1)	581 (0.2)		12 (0.1)
**Gender**							
Female	5126 (62.7)	3463 (53.9)	11128 (48.7)	116852 (25.8)	91655 (37.6)		9470 (43.3)
Male	3017 (36.9)	2956 (46.0)	11157 (48.9)	330563 (73.1)	151475 (62.2)		12306 (56.2)
Transgender/other	0 (0.0)	4 (0.1)	169 (0.7)	1210 (0.3)	94 (0.0)		20 (0.1)
Missing	29 (0.4)	7 (0.1)	376 (1.7)	3691 (0.8)	342 (0.1)		85 (0.4)
**Previous Testing**							
0–3 months	354 (4.3)	1092 (17.0)	690 (3.0)	81077 (17.9)	23639 (9.7)		2714 (12.4)
4–12 months	2500 (30.6)	2112 (32.8)	833 (3.7)	135779 (30.0)	68953 (28.3)		9313 (42.6)
> 12 months	2989 (36.6)	1446 (22.5)	559 (2.5)	69205 (15.3)	71441 (29.3)		6602 (30.2)
Never tested	1044 (12.8)	649 (10.1)	128 (0.6)	40409 (8.9)	19079 (7.8)		2357 (10.8)
Missing	1285 (15.7)	1131 (17.6)	20574 (90.2)	125846 (27.8)	60454 (24.8)		895 (4.1)

Nearly a quarter (*n* = 261 846, 24%) of all self-test kit recipients were adolescents and young people aged 15–24 years (Table 3). The sub-models with the highest proportions of 15–24 year-olds were transport hub (10%), hotspots (5%) and workplace (4%) (Table 4).

### Self-test kit recipients’ previous testing by model

Of those obtaining self-test kits and for whom we collected previous testing data (*n* = 771 612, 72%), 11% had never tested for HIV, 29% had not tested for at least a year, 41% had tested within the last 4–12 months and 19% had tested within the preceding three months (Table 3). Compared to the secondary (ANC and index testing) (4%) and key population (1%) models, the private sector, community-based and health facility OPD distribution approaches reached relatively high numbers of individuals with HIVST who had never tested for HIV (8% for all three models) (Table 3). Of note, key population and secondary (ANC and index testing) models had a high number of missing data for this variable (90% and 71%, respectively) (Table 3).

## Discussion

Our analysis of programme data for the period October 2017 to April 2020 has shown a high rate of HIV self-test kit distribution (> 1 million kits over 2.5 years) in South Africa across a variety of models and during routine service provision. These data add to the growing evidence on the feasibility and acceptability of this testing approach. The majority of kits (63%) were distributed through the community-based distribution model, mostly via the transport hub and hotspots sub-models, highlighting the practicality of using these approaches for further scale up.

Across models, 11% of self-test kit recipients for whom we collected previous testing data had never tested for HIV, highlighting the importance of HIV self-testing in reaching previously untested individuals, some of whom may not have tested otherwise [9]. While the proportion of first-time testers reached in this programme appears lower than reported in previous studies [9], this likely reflects how challenging reaching this group might become as countries are nearing achievement of global targets. When considering the high volume of kits distributed, however, this suggests that wide-scale HIVST programming continues to be an important strategy for SSA in general and South Africa, specifically in working towards achieving the first 95 of the 95–95-95 targets [1].

Data on models/sub-models that reach higher proportions of specific population groups will be critical in closing the HIV testing gap that remains, among men in general [9, 10] and key populations including sex workers and MSM [8]. Achieving greater coverage of diagnosis, treatment and viral suppression among men is not only important for their own health, but also reduces the risk of HIV transmission to female partners [3, 39]. HIVST strategies that reached men were a key focus of this programme and models that witnessed the highest number of kits distributed were transport hub, workplace and hotspots. These findings are unsurprising as the programme conducted specific outreach to men, and previous studies have reported similar results [9]. The workplace model implemented in this programme is now recognised as a particularly useful way to reach men [40, 41]. Given that men are recognised i) as constituting the greatest gap in HIV services in SSA in general and South Africa, specifically [2], ii) for avoiding health facilities [42], using a combination of non-facility-based testing approaches to reach this group is an urgent necessity [39].

We also explored the feasibility of reaching men with HIVST through their female sexual partners. A small proportion of males were reached with HIVST through female sex workers. This secondary distribution model has not yet been strongly demonstrated by programme data. Our data add to the growing evidence on how HIVST could be an effective tool to improve access to, and frequency of, HIV testing among sex workers’ clients and other sexual partners [33, 43, 44]. However, given the potential risks HIVST may pose to sex workers, who are particularly vulnerable to violence [31, 44] it is crucial to involve sex worker networks and communities in the design of these approaches.

Adolescents and young people (ages 15–24) continue to be an important group to be reached with HIV services including HIV testing in SSA. Here, six in seven new HIV infections among adolescents (aged 15–19 years) are among girls, and young women (ages 15–24) are twice as likely to be living with HIV than their male counterparts [8, 45, 46]. Further, adolescent girls and young women (AGYW, ages 15–24) accounted for 25% of HIV infections in 2020, despite representing just 10% of the population [8]. AGYW therefore need access to HIVST throughout periods of risk and, in the form of flexible delivery. Although we are unable to delineate the sub-models that were effective at specifically reaching AGYW, programme data showed HIVST’s utility in reaching adolescents and young people overall, with 24% of all distributions reaching this group. The sub-models with the highest proportions of 15–24 year-olds were transport hub, hotspots and workplace; these can be utilised going forward.

HIVST may be a critical entry point for other sexual and reproductive health and HIV services for young people, including family planning and pre-exposure prophylaxis (PrEP). WHO now recommends self-testing as an important tool for differentiated service delivery of PrEP (oral PrEP and Dapivirine Vaginal Ring) both to optimise delivery and enable more people to access PrEP [24]. Further implementation research is needed to guide programmes on how best to scale-up this strategy and measure its impact toward PrEP targets and the global goal to reduce annual new HIV infections to 370,000 by 2025 [18].

With a high number of distributed kits (> 1 million), we were able to make substantial inferences. A limitation is that self-test kit distribution approaches used were intentionally designed to target specific priority populations such as adult men who are generally less likely to have tested compared to women; thus, these findings should be considered in this context and other programmes without targeting may have different results. In addition, some sub-models had a high number of missing data for some variables, which made it difficult to derive certain inferences. Also, due to pragmatic reasons, we followed up the HIVST result for some clients/sub-models and not others, a factor which influences observed positivity rates. Finally, we did not explore the findings in-depth (e.g., qualitatively), which would have provided context and nuances.

## Conclusions

This analysis of programme data enabled us to identify self-test kit distribution models which could be used to reach men 20–35 years old and young people, groups at greatest risk of HIV acquisition in both South Africa and much of SSA. The data suggest that models/sub-models that reach targeted groups where they live, work and spend time, are likely to result in higher HIVST uptake. Study findings will be important in ensuring the scale-up of HIVST in South Africa is tailored to meet the needs of different population groups. Study findings are relevant to all programmes, government departments and implementers involved in scaling up HIVST.

## Data Availability

The datasets used and/or analysed during the current study are available from the corresponding author on reasonable request.

## Abbreviations

AGYW: Adolescent girls and young women
ANC: Antenatal care
ART: Antiretroviral therapy
FSW: Female sex worker
GP: General practitioner
HIV: Human immunodeficiency virus
HIVST: HIV self-testing
HTS: HIV testing services
MSM: Men who have sex with men
OPD: Outpatient department
PrEP: Pre-exposure prophylaxis
RDT: Rapid Diagnostic Test
SSA: Sub-Saharan Africa
STAR: Self-Testing AfRica
STI: Sexually Transmitted Infection
WHO: World Health Organisation
